# An Update Regarding the Use of Contemporary Dental Materials in Periodontal Regeneration

**DOI:** 10.3390/ma18184278

**Published:** 2025-09-12

**Authors:** Dragos Ioan Virvescu, Ovidiu-Sebastian Nicolaiciuc, Gabriel Rotundu, Florinel Cosmin Bida, Oana-Maria Butnaru, Zinovia Surlari, Mihaela Scurtu, Dana Gabriela Budala, Ionut Luchian

**Affiliations:** 1 Department of Dental Materials, Faculty of Dental Medicine, “Grigore T. Popa” University of Medicine and Pharmacy, 700115 Iasi, Romania; 2 Department of Dental Technology, Faculty of Dental Medicine, “Grigore T. Popa” University of Medicine and Pharmacy, 700115 Iasi, Romania; 3 Department of Periodontology, Faculty of Dental Medicine, “Grigore T. Popa” University of Medicine and Pharmacy, 700115 Iasi, Romania; 4 Department of Complete Dentures, Faculty of Dental Medicine, “Grigore T. Popa” University of Medicine and Pharmacy, 700115 Iasi, Romania; 5 Department of Biophysics, Faculty of Dental Medicine, “Grigore T. Popa” University of Medicine and Pharmacy, 700115 Iasi, Romania; oana.maria.butnaru@umfiasi.ro; 6 Department of Fixed Dentures, Faculty of Dental Medicine, “Grigore T. Popa” University of Medicine and Pharmacy, 700115 Iasi, Romania

**Keywords:** periodontal regeneration, dental biomaterials, guided tissue regeneration (GTR), bone grafts, enamel matrix derivatives, growth factors, scaffolds, stem cells

## Abstract

Background: Periodontal regeneration has become a focal point in modern dental therapy, aiming to restore the form and function of lost periodontal structures. A literature search was conducted on the PubMed, Scopus, and Web of Science databases, focusing on studies published between 2000 and 2025 that addressed the clinical use of dental biomaterials in periodontal regeneration. Emphasis was placed on the use of bone grafts, guided tissue regeneration (GTR) membranes, enamel matrix derivatives, scaffolds, growth factors, and stem cell-based technologies. The review also outlines the limitations of current strategies, including unpredictable clinical responses, the rapid degradation of bioactive components, and variability in healing. Emerging directions, such as nanotechnology, gene-activated matrices, and 3D-printed scaffolds, are highlighted for their potential to improve predictability and personalization in periodontal therapy. This synthesis underscores both the progress and ongoing challenges in the field, emphasizing the need for continued research into material innovation and patient-specific solutions.

## 1. Introduction

Periodontal disease is still one of the most common long-term inflammatory diseases that affect adults around the world. It slowly destroys the components that support the teeth, such as the gingiva, periodontal ligament, and alveolar bone [1]. Traditional periodontal treatments are successful in preventing disease development and managing inflammation, but they do not always work to repair lost tissues [2]. As a result, current periodontal therapy has moved towards regenerative techniques that aim to restore the periodontium both physiologically and functionally [3].

In the last few decades, a wide range of biomaterials have been used in periodontal regenerative materials. These include guided tissue regeneration (GTR) membranes, bone grafts (autografts, allografts, xenografts, and alloplasts), biologic mediators (such as enamel matrix derivatives and platelet-rich plasma/fibrin), and new scaffold systems that promote cell growth and tissue integration [4]. These materials have shown promising results in treating intrabony defects, furcation involvements, and recession-type lesions in both preclinical and clinical studies [5].

The perfect regenerative material would have to be able to pass a number of tests. It would have to be biocompatible to prevent cytotoxicity or immune rejection. It would also have to be osteoconductive to help progenitor cells to migrate and attach. It would also have to be osteogenic or osteoinductive to encourage cell differentiation towards a bone-forming lineage. Lastly, it would have to be space-maintaining to give a stable structure that allows for the selective repopulation of periodontal tissues [6]. Furthermore, it ought to facilitate tissue-specific healing, encourage quick vascularization, and blend in seamlessly with the host tissue, without disrupting regular cellular processes [7].

Bioactive and smart materials that can control the release of bioactive molecules or alter the host environment in real time have recently attracted a lot of interest [8,9]. New methods of enhancing surface characteristics, cellular interactions, and delivery systems for growth factors or gene therapy have also become possible thanks to nanotechnology [10,11]. Although it is still mostly in its experimental phase, stem cell therapy, in conjunction with scaffolds and signaling molecules, may one day completely restore function in a patient-specific manner [12,13].

This paper aims to provide a review of the current materials and approaches used in periodontal regenerative therapy, examining their biological rationale, clinical applications, efficacy, and limitations. By analyzing the available evidence, this work seeks to highlight both the progress and the challenges that define the current state of regenerative periodontology, while outlining potential future directions that may lead to more predictable and personalized treatment outcomes.

## 2. Literature Review

Using electronic databases including PubMed, Scopus, and Web of Science, a systematic search of the scientific literature was conducted for this narrative review. Key terms including “bone grafts”, “dental biomaterials”, “barrier membranes”, “enamel matrix derivatives”, “growth factors”, and “periodontal regeneration” were incorporated into the search approach. Priority was given to systematic reviews, randomized controlled trials, and pertinent experimental investigations reported in English among the articles published between 2000 and 2025.

Inclusion criteria:

✓ Articles addressing the use of dental biomaterials in periodontal regeneration.
✓ Human studies and in vivo or in vitro experimental research relevant to clinical application.
✓ Systematic reviews, meta-analyses, randomized controlled trials, cohort studies, and narrative reviews providing significant insights.
✓ Publications in English, published between 2000 and 2025.

Exclusion criteria:

✓ Studies focusing exclusively on non-periodontal applications (e.g., maxillofacial reconstruction unrelated to periodontium).
✓ Case reports or articles with insufficient methodological rigor.
✓ Animal studies without clear clinical translatability.
✓ Publications in languages other than English.
✓ Non-peer-reviewed literature (e.g., conference abstracts without full papers).

Relevant data were extracted manually and organized thematically to provide a comprehensive synthesis of current knowledge on the topic.

Our evolving knowledge of the biological and material science principles that enable tissue regeneration is demonstrated by studies pertaining to periodontal regeneration. The periodontium is a highly specialized unit of connective tissue that consists of four primary components: the alveolar bone, the cementum, the periodontal ligament (PDL), and the gingiva [14,15]. Restoring tissues to their pre-injury state in terms of structure, function, and biological integrity is the primary objective of periodontal regeneration treatment.

✓ Biological Principles of Periodontal Regeneration

Repair is the process of healing that creates a lengthy junctional epithelium or connective tissue scar. This restores the continuity of the tissue, but not always its original function or structure [16]. On the other hand, regeneration means restoring the lost periodontal structures, such as new cementum containing Sharpey’s fibers, a functionally orientated PDL, and new alveolar bone [17]. Connecting the teeth to the jawbone, the PDL plays a crucial role in maintaining their stability and health.

The PDL’s most important functions include anchoring the teeth and promoting the health of the gums and bone around them. The cells within it aid in the regeneration and repair of the tissues surrounding the teeth by creating substances that reinforce the bone and gums in the area [18]. Moreover, it protects the teeth and gums from damage, controls calcification, and encourages the growth of bone and cementum [19]. The fact that the PDL can regenerate is particularly notable.

The PDL contains cells that have the potential to differentiate into the specific cell types required for tooth and gum tissue healing, which promotes recovery and new growth [20]. The PDL contains stem cells that have the potential to repair periodontal tissues that have been injured, according to research [21]. Evidence of the PDL’s significance in contemporary tissue healing and potential medicinal treatments is the fact that new technologies, such as specialized scaffolds and membranes, are being created to improve the PDL and the surrounding bone’s regeneration even further [22].

**Figure 1:**
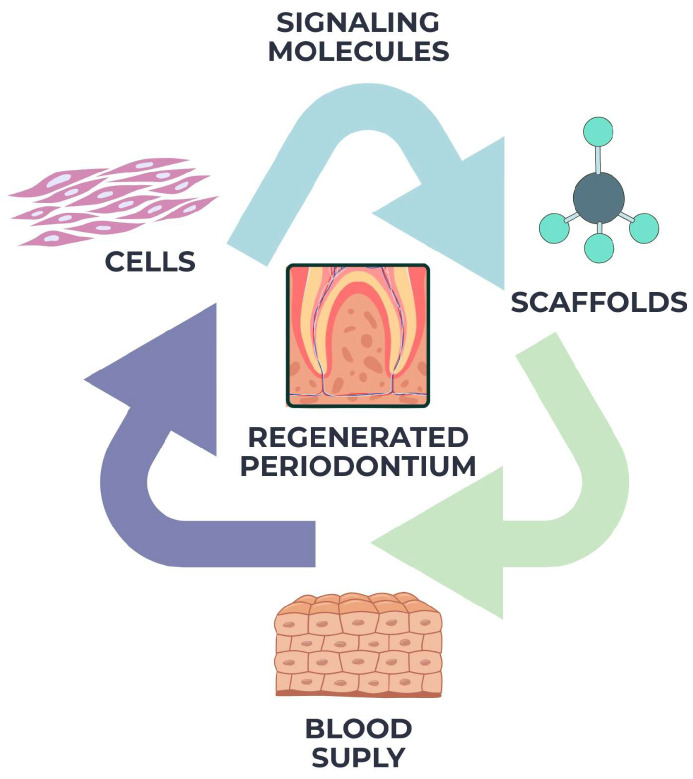
Principles of periodontal regeneration—schematic representation of the biological processes underlying periodontal regeneration, including cellular migration, proliferation, differentiation, and extracellular matrix deposition. The figure highlights the coordinated activity of periodontal ligament cells, cementoblasts, osteoblasts, and epithelial cells during tissue repair.

According to the rules of tissue engineering, cells, scaffolds, and signaling molecules must all work together in order for periodontal regeneration to be successful, as illustrated in Figure 1.

Stem cells, like mesenchymal stem cells from the periodontal ligament, gingiva, or bone marrow, can regenerate by transforming into important cell types like osteoblasts, cementoblasts, and fibroblasts [23].

A three-dimensional scaffold acts as a supportive matrix that allows cells to stick to each other, grow, and organize themselves in space. Bioactive signals, such as growth factors like platelet-derived growth factor (PDGF), bone morphogenetic proteins (BMPs), transforming growth factor beta (TGF-β), and insulin-like growth factor (IGF), control cellular activities and guide the healing process by encouraging chemotaxis, proliferation, and differentiation [24,25].

In the periodontal context, space maintenance and wound stability are also critical prerequisites. Because the oral environment is dynamic and prone to bacterial contamination, regenerative outcomes are highly sensitive to disturbance [26]. Membranes used in guided tissue regeneration (GTR) are designed to provide a barrier that allows selective repopulation by the PDL and bone cells, while excluding faster-proliferating epithelial cells. Furthermore, angiogenesis plays a key role in regeneration by ensuring adequate nutrient delivery and immune regulation. Emerging evidence suggests that vascular endothelial growth factor (VEGF) and other pro-angiogenic signals are essential for successful periodontal wound healing [27,28].

In summary, periodontal regeneration is a highly orchestrated biological process that depends on the availability and interactions of appropriate cells, scaffolding materials, and bioactive signals. A comprehensive understanding of these principles is essential in selecting or designing regenerative strategies that can reliably reestablish periodontal structure and function.

✓ Classification of Regenerative Materials

In periodontal regeneration, a broad range of biomaterials have been created and used, each with its own unique function in facilitating the recovery of damaged tissues. Each of these materials has its own unique history, composition, biological characteristics, and medical uses [29]. Bone grafts, barrier membranes, biologic mediators (e.g., growth factors, enamel matrix derivatives), scaffold systems, autologous platelet concentrates, and, more recently, stem cell-based techniques and cutting-edge technologies (e.g., nanomaterials, 3D printing) are all categorized into this group [30].

Each category contributes uniquely to the regeneration process—either by supplying a structural framework, promoting cell proliferation and differentiation, or improving biological environments [31]. The choice of a particular material or its combination is based upon criteria like the defect shape, patient condition, and intended therapeutic outcome. The main categories of regenerative materials used in periodontal therapy are summarized in Table 1 below.

Biomaterials used in periodontal regeneration can be positioned within the generational classification framework. Traditional bone graft substitutes and non-resorbable membranes largely reflect second-generation bioactive materials, designed to support osteoconduction and barrier function. Enamel matrix derivatives and bioactive agents represent third-generation approaches, as they actively stimulate cellular responses and tissue regeneration.

More advanced scaffolds functionalized with growth factors or stem cells, as well as smart polymers with controlled release properties, correspond to fourth- and emerging fifth-generation biomaterials. Thus, the current clinical practice in periodontology mainly employs second- to third-generation biomaterials, while translational research is progressively advancing toward fourth- and fifth-generation solutions.

- Bone grafts

Bone grafts are among the most recognized and extensively utilized materials in periodontal regeneration, principally owing to their ability to restore bone volume and facilitate the reattachment of periodontal structures [32]. They are mainly classified as autografts, allografts, xenografts, and alloplasts [32].

Their regenerative capacity depends on three primary biological mechanisms: the process known as osteoinduction initiates new bone production by stimulating mesenchymal stem cells to develop into osteoblasts. In osteogenesis, osteoblasts play a mediating role in the process of bone production. In order to build new bone, osteoconduction acts as a scaffold by allowing the incorporation of neighboring bone [39]. Autografts have two potential uses: fresh and frozen. Root resorption is more common after using fresh autografts as opposed to frozen ones. Clinical investigations and systematic reviews have repeatedly shown the effectiveness of bone grafts in treating intrabony defects, class II furcation lesions, and alveolar ridge preservation treatments [40].

Although autografts are regarded as the gold standard because of their intrinsic cellular activity and potential for integration, their application is constrained by the necessity of a secondary surgical site and associated donor site morbidity [40].

Clinical practice frequently employs human allogeneic bone transplant materials as autogenous bone replacements. Allogeneic grafts are similar to autografts in some ways, but they lack cells that produce bone because decalcification and freeze-drying remove these cells from the tissue. There is no need to find a donor location, they can be utilized in greater amounts as needed, and they are more biologically equivalent to natural human tissue than xenografts or synthetic alternatives [41,42]. Nevertheless, there are a number of drawbacks to these materials that should be considered. These include the possibility of infection, a lack of osteoinductive ability, an extended healing period for bone regeneration, and the possibility of immunological reactions caused by persistent antigens that may remain after processing [43].

As a result, xenografts like deproteinized bovine bone (e.g., Bio-Oss) and synthetic alloplasts have been more favored because of their accessibility, biocompatibility, and ease of manipulation, despite their slower resorption rates and diminished osteogenic potential [44]. Current research emphasizes improving the biological efficacy of grafts using composite formulations, the integration of bioactive compounds, and cell-seeding methodologies, with the objective of boosting predictability and expediting integration in complex clinical situations [45,46].

- Guided tissue regeneration (GTR) membranes

Guided tissue regeneration membranes are essential in periodontal regenerative therapy, serving as selective barriers that inhibit the swift migration of epithelial cells into periodontal defects, thus permitting the gradual repopulation of the site by periodontal ligament and bone cells [47]. The fundamental biological concept relies on cell exclusion, a process that promotes tissue-specific regeneration when an appropriate healing environment is sustained [48,49].

Two primary categories of membranes are utilized: resorbable membranes, often constructed from collagen or synthetic polymers (e.g., polylactic acid, polycaprolactone, polylactic-co-glycolic acid), and non-resorbable membranes, such as those created from expanded polytetrafluoroethylene (ePTFE), high-density polytetrafluoroethylene (d-PTFE), titanium-reinforced PTFE, and titanium mesh [50]. Synthetic polymers like these make it possible to customize important qualities like mechanical strength, porosity, and the degradation rate to specific therapeutic needs, while also allowing for exact control over the composition and scalable production. On the other hand, they have weak cell-binding abilities, which can hinder cell adhesion, and they may be toxic or immunogenic [51,52].

Mechanical strength, biocompatibility, and the ability to assist cellular and bone regeneration are key considerations when choosing a membrane type [50,51,52].

Resorbable membranes are preferred for their biocompatibility and the obviation of further surgical procedures; however, their structural integrity may be compromised in larger or more intricate lesions [53].

Non-resorbable membranes provide superior space preservation and mechanical stability; nevertheless, they necessitate surgical removal and exhibit an increased risk of bacterial contamination upon exposure [54]. The choice between resorbable and non-resorbable membranes depends on several critical factors, including the composition, mechanical performance, and clinical handling. Table 2 summarizes the main characteristics, advantages, and limitations of each membrane type based on current evidence.

Clinical studies validate the efficacy of GTR membranes, especially when utilized alongside bone grafts, in facilitating periodontal regeneration in intrabony and class II furcation deficiencies [55]. Results from clinical trials comparing open flap debridement alone to clinical attachment growth and probing pocket depth reduction showed that biomaterials and barrier membranes used together were superior [56,57,58].

**Figure 2:**
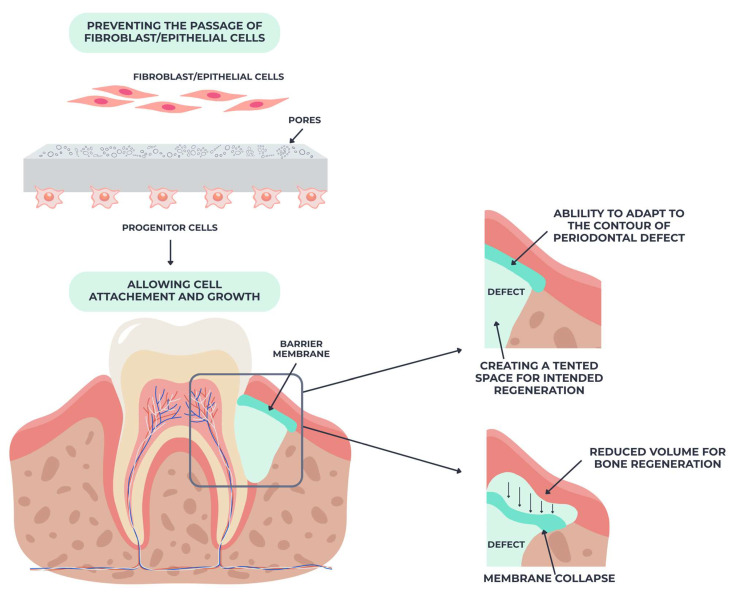
Guided tissue regeneration membranes—the membrane prevents epithelial and fibroblast migration into the defect, while allowing periodontal progenitor cells to attach and proliferate. Adequate membrane stability maintains a protected space for bone and periodontal regeneration, whereas membrane collapse reduces the regenerative potential.

To prevent fibroblasts and epithelial cells from migrating into the defective location, the barrier membrane selectively allows progenitor cells to enter the area beneath the membrane. Cell adhesion and development are facilitated by this selective permeability, which aids in tissue healing, as pictured in Figure 2.

Compared to open flap debridement alone, intrabony defects treated with GTR had better clinical attachment growth, a probing pocket depth decrease, and a gingival recession increase in the meta-analysis and systematic review by Murphy and Gunsolley [59] and Needleman [60], respectively. Trombelli observed that GTR improved clinical attachment and probing pocket depth changes and boosted defect filling compared to open flap debridement alone in another comprehensive review [61]. These evaluations demonstrate the many benefits of biomaterials, but they also point out problems in determining the therapeutic significance of the results and the fact that there is broad variability among research.

Moreover, problems including membrane exposure and premature degradation may jeopardize therapeutic results. Recent advancements include improving membrane efficacy through crosslinking methods, the inclusion of antibacterial substances or growth hormones, and the creation of multilayered or biomimetic membranes that more accurately replicate the natural extracellular matrix, hence promoting integration and healing [62,63].

The influence of and ongoing improvements in new technologies allow for the proposal of new lines of inquiry, such as the use of artificial intelligence (AI) in membrane design to optimize the tailoring of mechanical properties, enable controlled degradation, and enhance the pore structures of membranes in order to enhance their performance.

- Growth factors

Growth factors play a central role in orchestrating the complex cascade of cellular events required for periodontal regeneration. These bioactive proteins act as signaling molecules that regulate key biological processes such as chemotaxis, proliferation, differentiation, and matrix synthesis in target cells. In periodontal applications, the most commonly studied growth factors include platelet-derived growth factor (PDGF), bone morphogenetic proteins (BMPs), transforming growth factor beta (TGF-β), insulin-like growth factor (IGF), and fibroblast growth factor (FGF) [64].

Administered locally, either in isolation or incorporated into biomaterials such as scaffolds or membranes, these molecules can significantly enhance regenerative outcomes by promoting neovascularization and the recruitment of progenitor cells to the defect site [65].

According to clinical research, growth factor-enhanced therapies can improve bone filling, attachment gains, and healing in intrabony and furcation defects, particularly when combined with guided tissue regeneration membranes or bone transplants [66]. Nonetheless, obstacles such as fast degradation, explosive release kinetics, and financial constraints have impeded their regular clinical use.

Current research is focused on controlled release methods, gene-activated matrices, and multifactorial combinations designed to emulate the natural signaling gradients of wound healing while maintaining localized and prolonged bioactivity [67]. Notwithstanding encouraging outcomes, further long-term, rigorously controlled clinical trials are requisite to formulate standardized methods and enhance treatment effectiveness.

Autologous platelet concentrates have received considerable attention in periodontal regeneration due to their abundant growth factors and cytokines, sourced directly from the patient’s blood [68]. These biomaterials, combining platelet-rich plasma (PRP), platelet-rich fibrin (PRF), and concentrated growth factors (CGFs), function as natural scaffolds and biological enhancers that facilitate wound healing, angiogenesis, and the regeneration of soft and hard tissues [69].

In contrast to synthetic growth factor applications, platelet concentrates release a diverse array of bioactive molecules in a physiological way, including PDGF, TGF-β, VEGF, and IGF, which regulate inflammation and enhance cellular activity at the defect site [70]. PRF, a second-generation product, possesses the benefit of creating a fibrin matrix that progressively releases these components over several days, eliminating the necessity of anticoagulants or chemical additions [71].

**Figure 3:**
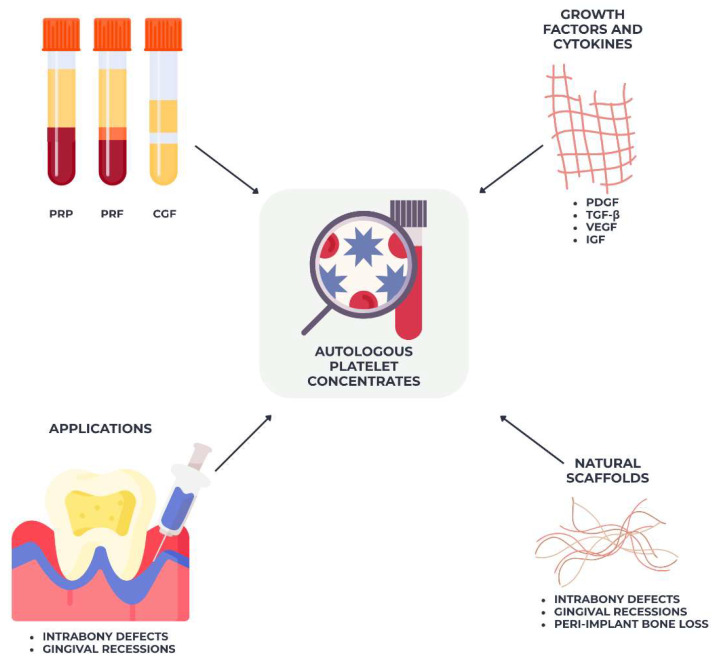
Platelet concentrates and their role in periodontal tissue engineering—platelet-rich plasma (PRP) and platelet-rich fibrin (PRF) act as autologous reservoirs of growth factors (PDGF, TGF-β, VEGF), promoting angiogenesis, fibroblast proliferation, and new bone formation. They can be combined with grafts or membranes to accelerate tissue regeneration.

Platelet concentrates have been utilized independently or with bone grafts and membranes in the management of intrabony defects, gingival recessions, and peri-implant bone loss, frequently demonstrating enhanced healing dynamics and accelerated tissue maturation [69], as shown in Figure 3 Nonetheless, their regeneration effectiveness is inconsistent and significantly influenced by individual patient characteristics, the blood processing method, and the defect form [68].

Potential negative effects must also be taken into account, even if growth factors like PDGF, TGF-β, and BMPs are essential in promoting cell proliferation, angiogenesis, and differentiation [72]. Fibrosis, ectopic calcifications, and aberrant tissue reactions can result from excessive or uncontrolled administration. Furthermore, there is a chance that growth factor signaling could encourage malignant cell transformation in severely damaged oral mucosae with dysplastic alterations by boosting the proliferation of genetically unstable cells [72]. Consequently, cautious patient selection, regulated distribution methods, and ongoing monitoring are necessary for the clinical application of growth factors [73].

Table 3 shows the results of several research works that have examined the use of recombinant growth factors for oral tissue regeneration, either alone or in combination with other growth factors and biomaterials.

Growth factor delivery in vivo is now more effective thanks to the creation of new delivery methods. In order to sequester growth factors and release them at optimal doses in a timely manner based on the biological demands of the target tissue, bioabsorbable controlled-release scaffolds have been developed [83]. Numerous investigations have demonstrated that growth factors can be released for up to 15 days when linked to microspheres that contain poly(lactate-co-glycolide) scaffolds [84,85].

Because they are prepared from the patient’s own blood, platelet-rich plasma (PRP) and platelet-rich fibrin (PRF) are highly customized therapeutic resources [86]. In addition to their inherent regenerative properties, these concentrates have the ability to improve tissue repair and speed up periodontal regeneration when coupled with bone transplants, membranes, or scaffolds [87]. Better clinical results are the result of combinatorial techniques that use growth factors’ biological action in conjunction with biomaterials’ structural support.

Despite their regenerative potential, the clinical outcomes of autologous platelet concentrates such as PRP and PRF remain highly variable. This variability is largely attributable to patient-specific factors, including differences in baseline platelet counts, systemic health status, age, and lifestyle influences, all of which directly affect the concentrations and bioactivity of the growth factors released [86].

Moreover, systemic conditions such as diabetes or smoking can impair the biological activity of platelet-derived products, further reducing their predictability. Variability is also introduced by the different centrifugation protocols, devices, and handling techniques employed across studies, which result in heterogeneity in the fibrin architecture, leukocyte content, and release kinetics of bioactive molecules [86,87].

As a result, while platelet concentrates may offer substantial benefits in selected cases, the absence of standardized preparation methods and the strong influence of individual patient biology remain major challenges that limit their widespread clinical application.

The current state of affairs necessitates further investigation into the development of multimodal delivery systems capable of the controlled release of growth factors over time and space. One way to improve the efficacy of growth factor therapy in regenerating cells is to use gene-activated matrices, nanocarriers, or smart biomaterials that can adapt to their surrounding environments.

If we wish to standardize clinical procedures, verify safety and cost-effectiveness, and identify patient-related variables that impact treatment outcomes, we need large-scale randomized controlled trials with long-term follow-up. The future of periodontal tissue engineering may lie in a patient-specific regeneration approach that integrates biomarker evaluation with the development of scaffolds tailored to each individual.

- Enamel matrix derivates

A biologically inspired method of periodontal regeneration is represented by enamel matrix derivatives (EMDs), which are sourced from proteins found in pig tooth enamel [88]. Their main function is to encourage the regeneration of important periodontal structures, like cementum, the periodontal ligament, and alveolar bone, by imitating the normal processes of root formation, especially cementogenesis [89].

Emdogain, which is mostly composed of amelogenins contained in a propylene glycol alginate carrier, is the most extensively researched and therapeutically utilized product in this category [90]. Following debridement, EMD can be applied to the root surface to encourage PDL cell growth while blocking epithelial cell migration. This creates an ideal setting for new attachment formation [91].

In both intrabony and class II furcation lesions, EMD considerably improves clinical attachment gains and defect filling, according to many meta-analyses and randomized controlled studies [92,93]. It accelerates soft tissue recovery and lessens postoperative pain, and it is highly beneficial when combined with less invasive surgical procedures [94,95].

Its use is easy and well tolerated, but it has several drawbacks, such as a greater cost compared to more traditional treatments and a less consistent reaction in large or non-contained abnormalities [96]. Research focuses on combining EMD with bone grafts, platelet concentrates, and functionalized membranes to optimize regenerative outcomes in more challenging clinical cases [97,98,99,100].

- Scaffolds

Periodontal tissue engineering relies heavily on scaffolds, which facilitate cellular adhesion, migration, proliferation, and differentiation by offering a three-dimensional framework [101]. These structures, which function as temporary matrices, aid in the spatial organization of regenerated tissues and make it easier for nutrients and blood vessels to ingress and diffuse.

Given their substantial influence on the biological reactions of tissue cells and the ensuing creation of tissue, biomaterial scaffolds are acknowledged as an essential part of tissue engineering [101,102]. Improving periodontal regeneration applications is possible with bioactive scaffolds because they stimulate the differentiation and osteogenic/cementogenic gene expression of periodontal ligament cells (PDLCs), in contrast to traditional barrier biomaterial approaches that inhibit the downward growth of connective and epithelial tissues into the damaged site [103].

**Figure 4:**
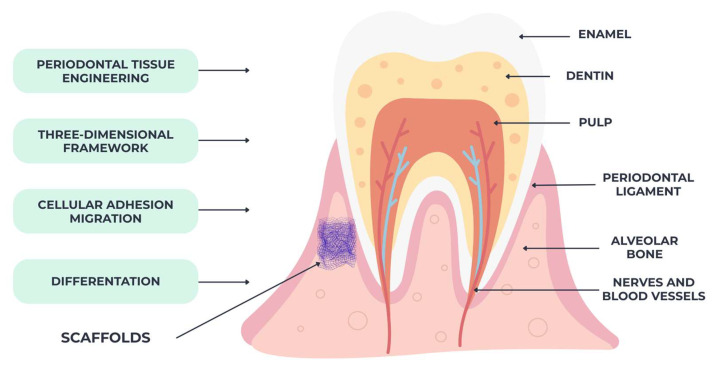
Functional role of scaffolds in periodontal regeneration—scaffolds provide a three-dimensional framework that supports cell adhesion, proliferation, and differentiation. Their porosity and biodegradability enable vascular ingrowth and tissue integration, while also serving as carriers for stem cells and bioactive molecules to enhance regeneration.

As illustrated in Figure 4, scaffolds facilitate the restoration of periodontal tissues by creating a microenvironment conducive to cell–matrix interactions and tissue remodeling.

Natural scaffolds include collagen, fibrin, and chitosan; synthetic scaffolds include polylactic-co-glycolic acid [PLGA] and polycaprolactone [PCL]; and composite scaffolds combine elements of both types to achieve a balance between biocompatibility and mechanical stability. Biocompatible, slowly biodegradable, and structurally supporting scaffolds are ideal for the targeted regeneration of alveolar bone, the periodontal ligament, and cementum [104].

The incorporation of controlled drug delivery systems, functionalization with growth factors, and nanostructured surfaces are all examples of recent developments in scaffold design that improve their bioactivity and integration [104]. A number of potential benefits may result from incorporating bioactive molecules into scaffolds. These include increased stability and longer storage times, reduced immunogenic potential, easier production and isolation processes, the ability to pass through biological barriers, and the targeted delivery of regenerative factors. Additionally, there is a reduced risk of tumorigenicity [103,104].

The use of 3D printing technology also makes it possible to tailor the structures of scaffolds to the specific shapes of defects [105]. An exciting new development in 3D printing is the use of bio-inks, which contain live cells and bioactive compounds. These inks allow for the integration of growth factors and signaling molecules, as well as the precise control of the pore size, permeability, and porosity. Fabricated scaffolds have been further improved by recent advances in 3D printing technology, which include the optimization of bio-inks, printing with multiple materials, and the incorporation of advanced imaging technologies for patient-specific scaffold design [106].

Modern technology enables the development of scaffolds that may be fine-tuned in terms of mechanical qualities, degradation rates, and the controlled release of therapeutic chemicals, all of which contribute to better regenerative results. Scaffolds that are structurally sound and that encourage cell proliferation, differentiation, and tissue regeneration are necessary to address the significant challenge of recreating the intricate compositions of these tissues [107,108].

- Stem cells

The remarkable capacity of stem cells to self-renew and specialize into various cell types is a significant advantage in periodontal regeneration, as it allows for the reconstruction of damaged periodontal tissues. Mesenchymal stem cells (MSCs) have been extensively researched from several sources, including bone marrow, gingival tissue, dental pulp, and the PDL [109].

These cells have the ability to produce osteogenic, cementogenic, and fibroblastic tissues. The regeneration potential of these cells can be amplified by combining them with growth factors and biocompatible scaffolds before delivery to the site of the injury [110]. The results of stem cell-based treatments for alveolar bone growth, periodontal attachment, and functional ligament reformation have been promising in preclinical research [111].

Another promising cell-free option that is able to modulate inflammation and stimulate tissue healing through paracrine pathways is exosomes produced from stem cells. Regulatory restrictions, expenses, a lack of protocol standardization, and difficulties in obtaining cells are the key reasons that these advancements have not yet found widespread clinical use [112].

In addition to bone marrow and adipose-derived stem cells, the oral cavity provides multiple accessible sources of mesenchymal stem cells. Stem cells from exfoliated deciduous teeth are characterized by a high proliferative rate and multipotent differentiation ability [113]. Dental pulp stem cells exhibit strong odontogenic and neurogenic potential, while stem cells from the apical papilla are particularly effective in root development and dentin formation [114]. Periodontal ligament stem cells contribute to cementum and ligament regeneration, and dental follicle stem cells have been shown to differentiate into periodontal and osteogenic lineages [37]. The relative ease of harvesting and their regenerative versatility make these cell populations valuable candidates for translational applications in periodontal and craniofacial tissue engineering.

Stem cell-based therapies represent a promising frontier in periodontal regeneration due to their capacity for self-renewal, multilineage differentiation, and immunomodulatory effects [115,116]. Although preclinical outcomes are encouraging, large-scale clinical trials are still required to validate their long-term efficacy and optimize treatment protocols [117]. The convergence of stem cell science, nanotechnology, and biofabrication holds the potential to redefine periodontal regenerative therapy in the coming decades.

## 3. Advances and Future Trends

Although there has been a great deal of progress in creating and using biomaterials for periodontal regeneration in the clinic, there are still certain problems that need to be solved. The future of this field depends significantly on new developments in tissue engineering, biomolecular signaling, and personalized regenerative techniques.

One interesting strategy is to combine stem cell-based therapies, especially mesenchymal stem cells and their derivatives, which can boost the regeneration capacity by changing the immune system and sending signals to other cells. Their combination with scaffolds and signaling molecules may lead to more consistent and predictable outcomes.

Using smart biomaterials that react to inflammation and enzymatic activity, which are local biological cues, bioactive chemicals can be regulated and delivered to the site of periodontal disorder. By reducing possible side effects, these materials have the potential to significantly improve the efficacy of treatments.

The use of 3D printing technology has the potential to revolutionize scaffold design by enabling the creation of one-of-a-kind structures tailored to the specific anatomical and functional requirements of each defect.

Potentially useful applications of nanotechnology include enhancing medication delivery precision, cell adhesion, and scaffold surface quality. Periodontal repair could be accelerated and its efficacy improved if this happens.

The heterogeneity of the reported outcomes in periodontal regeneration is partly explained by the fact that biomaterials often perform differently depending on the defect morphology. For example, bone graft substitutes and GTR membranes tend to achieve more predictable results in contained intrabony defects, where the natural bony walls facilitate clot stabilization, space maintenance, and vascular supply [118]. These structural advantages provide an ideal environment for graft integration and tissue regeneration [119].

In contrast, furcation defects remain particularly challenging due to their anatomical complexity, reduced vascularization, and limited accessibility for thorough debridement and biomaterial placement [120]. The presence of root concavities and the bifurcation anatomy often compromise space maintenance and increase the risk of bacterial recolonization. Consequently, outcomes in furcation therapy are less predictable and frequently less favorable compared with intrabony sites [121].

It is crucial to customize regenerative techniques according to the specific defect morphology when choosing and using biomaterials, as these disparities in clinical performance explain the contradictory findings in the literature.

Finally, before new materials and technologies are widely employed, it is vital to confirm them through long-term, multicenter trials and standardized clinical protocols. A shift in policy is necessary to ensure that new regenerative solutions are accessible, inexpensive, and safe.

## 4. Conclusions

Periodontal regeneration is still a primary goal of modern periodontology. The goal is to restore not only the structure but also the biological function of the destroyed periodontal tissues. In recent years, there have been major improvements in the creation and use of different biomaterials in the clinic.

This review has highlighted the biological principles that underpin periodontal regeneration, such as osteoconduction, osteoinduction, and cell signaling, alongside the clinical performance of different material classes. While the current materials have demonstrated varying degrees of success in promoting periodontal healing, limitations such as variable outcomes, immune responses, and a lack of long-term predictability persist.

The use of stem cell therapy in conjunction with nanomaterials and 3D-printed scaffolds might greatly improve regeneration results. Before these new technologies are incorporated into clinical practice routinely, however, more comprehensive clinical trials and efforts to standardize them are required.

## Figures and Tables

**Table 1:** Regenerative materials in periodontal therapy: classification and roles.

Category	Subtypes	Main Functions
Bone grafts	Autografts, allografts, xenografts, alloplasts	Osteoconduction, osteoinduction [32]
GTR membranes	Non-resorbable, resorbable	Guided tissue regeneration, barrier function [33]
Growth factors	Platelet-derived growth factor (PDGF), bone morphogenetic proteins (BMPs)	Stimulate cellular proliferation, differentiation, matrix synthesis [34]
Enamel matrix derivatives	Emdogain	Promote cementogenesis and periodontal ligament regeneration; stimulate fibroblasts and osteoblasts [35]
Platelet concentrates	Platelet-rich plasma (PRP), platelet-rich fibrin (PRF)	Enhance soft and hard tissue healing [36]
Scaffolds	Synthetic polymers/natural polymers	Support cell adhesion and proliferation [37]
Stem cells	Mesenchymal stem cells (MSCs), periodontal ligament stem cells (PDLSCs)	Promote regeneration of periodontal tissues [38]

**Table 2:** Comparison between resorbable and non-resorbable membranes in GTR.

Characteristic	Resorbable Membranes	Non-Resorbable Membranes
Composition	Collagen, synthetic polymers (e.g., polylactic acid)	Expanded polytetrafluoroethylene (ePTFE)
Biocompatibility	High	High
Need for Second Surgery	Not required	Required for removal
Mechanical Strength	Lower—may be insufficient in large or complex defects	Higher—excellent space maintenance
Risk of Bacterial Contamination	Lower	Higher upon membrane exposure
Application Preference	Preferred in routine cases due to resorption and ease of use	Preferred in cases requiring enhanced space stability

**Table 3:** Preclinical and clinical studies using growth factors in oral and periodontal healing.

Growth Factor	Preclinical Studies	Clinical Studies
BMP-2	Periodontal defects: alveolar bone formation [74] Mandibular bone defects: stimulating the formation of new bone [75]	Alveolar bone: promoted bone ridge augmentation [76]
BMP-7	Enhanced cementum and bone repair in periodontal furcation deficiencies [77]	
BMP-12	Periodontal defects: new PDL with improved fiber orientation when compared with BMP-2 [78]	
FGF-2	Furcation and intrabony periodontal defects: improved periodontal regeneration (new bone, cementum, and PDL) [79]	
PDGF	Periodontal furcation and fenestration defects: accelerated healing and improved PDL and bone regeneration [80]	Furcation and intrabony defects: histological evidence of periodontal regeneration in proof-of-principle studies [81]; improved clinical attachment level gain and radiographic bone filling [82]
TGF-β	Periodontal defects: insignificant improvement in cementum formation; conflicting results regarding alveolar bone regeneration [83,84]	

## Data Availability

No new data were created or analyzed in this study. Data sharing is not applicable to this article.

## Abbreviations

The following abbreviations are used in this manuscript:

GTR	guided tissue regeneration
PDL	periodontal ligament
PDGF	platelet-derived growth factor
BMPs	bone morphogenetic proteins
TGF-β	transforming growth factor beta
IGF	insulin-like growth factor
VEGF	vascular endothelial growth factor
ePTFE	expanded polytetrafluoroethylene
PLGA	polylactic-co-glycolic acid
PCL	polycaprolactone
PRP	platelet-rich plasma
PRF	platelet-rich fibrin
CGF	concentrated growth factor
EMDs	enamel matrix derivatives
MSCs	mesenchymal stem cells
OFD	open flap debridement
